# Transcriptome Analysis of Pineal Glands in the Mouse Model of Alzheimer’s Disease

**DOI:** 10.3389/fnmol.2019.00318

**Published:** 2020-01-09

**Authors:** Kwang Il Nam, Gwangho Yoon, Young-Kook Kim, Juhyun Song

**Affiliations:** ^1^Department of Anatomy, Chonnam National University Medical School, Jeollanam-do, South Korea; ^2^Department of Biochemistry, Chonnam National University Medical School, Jeollanam-do, South Korea; ^3^Department of Biomedical Sciences, Center for Creative Biomedical Scientists at Chonnam National University, Jeollanam-do, South Korea

**Keywords:** pineal gland, Alzheimer’s disease, long noncoding RNA, circular RNA, RNA sequencing

## Abstract

The pineal gland maintains the circadian rhythm in the body by secreting the hormone melatonin. Alzheimer’s disease (AD) is the most common neurodegenerative disease. Pineal gland impairment in AD is widely observed, but no study to date has analyzed the transcriptome in the pineal glands of AD. To establish resources for the study on pineal gland dysfunction in AD, we performed a transcriptome analysis of the pineal glands of AD model mice and compared them to those of wild type mice. We identified the global change of diverse protein-coding RNAs, which are implicated in the alteration in cellular transport, protein transport, protein folding, collagen expression, histone dosage, and the electron transfer system. We also discovered various dysregulated long noncoding RNAs and circular RNAs in the pineal glands of mice with AD. This study showed that the expression of diverse RNAs with important functional implications in AD was changed in the pineal gland of the AD mouse model. The analyzed data reported in this study will be an important resource for future studies to elucidate the altered physiology of the pineal gland in AD.

## Introduction

Alzheimer’s disease (AD), the most common neurodegenerative disease worldwide, is characterized by progressively impaired cognition, an excessive accumulation of amyloid-β (Aβ), and abnormally hyperphosphorylated tau in the brain (Jack et al., 2013; Song, 2019). It was reported that AD patients showed calcification of the pineal gland and reduced melatonin levels in the serum and cerebrospinal fluid (Skene and Swaab, 2003). Reduced pineal gland volume was also observed in AD patients (Mahlberg et al., 2008). Moreover, a decreased melatonin level is correlated with cognitive impairment (Srinivasan et al., 2010; Rosales-Corral et al., 2012). Although these studies showed causality, however, there is still controversy on the role of pineal gland and melatonin in AD because no clear mechanism has yet been identified.

The pineal gland, which contains neuroglial cells and predominantly pinealocytes, is one of the central organs that regulate the circadian system and is innervated by a neural synaptic pathway originating in the suprachiasmatic nucleus within the hypothalamus (Simonneaux and Ribelayga, 2003). The pineal gland also acts as a modulator in the sexual maturation and aging process as well as sleep disturbance (Bumb et al., 2014). Melatonin is synthesized and secreted in the pineal gland, and its secretory capacity is significantly proportional to pineal parenchymal volume (Nölte et al., 2009). Melatonin contributes to improved hippocampal neurogenesis in AD (Sarlak et al., 2013) and protects neurons against death induced by oxidative stress (Shukla et al., 2017) and Aβ toxicity (He et al., 2010). Pineal dysfunction and reduced melatonin levels are directly related to the pathological progression of AD (Wu et al., 2003). The reduced volume and calcification of the pineal gland influences its function; ultimately, it is strongly associated with the diverse neuropathology of AD patients. However, the mechanisms connecting pineal gland dysfunction and AD pathologies are not fully understood. Thus, more detailed analyses on the molecular level are required to identify the relationship between the pineal gland and AD.

Current annotation of the human genome shows that approximately 90% of the human genome is transcribed, 3% of the genome comprises protein-coding genes, and the rest is noncoding RNA (Harrow et al., 2006). Noncoding RNAs modulate gene expression and are divided into two subclasses according to their length. Long noncoding RNAs (lncRNAs; >200 nt in general) are of special issue owing to their large numbers and the possibility that they are functionally crucial components of the genome (Tsai et al., 2010). Many lncRNAs are associated with epigenetic processes affecting gene expression. The expression of lncRNAs could be regulated by the transsynaptic/cAMP system that controls the expression of hundreds of protein-coding genes in the pineal gland (Coon et al., 2012). CircRNA as a different class from other noncoding RNAs is produced by the back-splicing of a single-stranded linear transcript. In the central nervous system, some circRNAs have a regulatory role in synaptic plasticity induction in neurons (You et al., 2015). Although many noncoding RNAs are linked with the neurological function and identified in the pineal gland, the roles of noncoding RNAs in the AD pineal gland has not been investigated until now.

Here we analyzed diverse RNAs altered in the pineal gland of AD and investigated the function of those RNAs related to the pineal gland in the AD brain. We expect that our analysis of the identification and functional analysis of the transcriptome in the AD pineal gland may be an important resource to solve the neurological interaction between the pineal gland and AD pathogenesis.

## Materials and Methods

### Sample Preparation

We obtained male 5xFAD transgenic mice [B6.Cg-Tg (APPSwFlLon, PS1*M146L*L286V) 6799Vas/Mmjax; JAX MMRRC stock number: 34848] from The Jackson Laboratory (Bar Harbor, ME, USA). Aβ_42_ production was identified in the entire brain at 2 months. Wild-type male mice (C57BL/6) were purchased from Koatech (Pyeongtaek, South Korea). For pineal gland harvesting, the mice with 5 months of age were sacrificed under ether anesthesia. We took samples from all mice at the same time of the day [Zeitgeber time (ZT) 0.5] under conditions of 12 h in light and 12 h in dark. There was no noticeable difference in the phenotype between the pineal glands of wild type and 5xFAD mice. The pineal glands from five to seven mice were pooled in each sample, and four samples in each of wild type and 5xFAD group were prepared for the analysis. The experiment was performed in accordance with the recommendations of “96 Guidance for Animal Experiments” established by the Animal Ethics Committee at Chonnam National University. The experimental protocols were approved by the Animal Ethics Committee at Chonnam National University.

### RNA Sequencing

Total RNA from the pineal gland was extracted using TRIzol reagent (Thermo Fisher), and their integrity was checked using the Agilent 2100 BioAnalyzer (Agilent). Total RNA was treated with a Ribo-Zero Gold rRNA Removal Kit (Illumina) to remove ribosomal RNAs, and RNA sequencing libraries were constructed using a TruSeq Stranded Total RNA Kit (Illumina). The libraries were paired-end sequenced with 100 sequencing cycles on a HiSeq 2500 system (Illumina).

### Analysis of RNA Sequencing Data

By using FastQC, we assessed the quality of our sequencing data. When the median value of per base sequence quality was calculated from all sequencing libraries, the quality was shown to be very high across all nucleotides. The overall quality was satisfactory in all libraries as calculated from the high percentage of nucleotides with a quality score above 30 (Table 1).

**Table 1:** Sample information by nucleotide number and Phred quality score.

Name	Total reads	Total bases	GC rate	N rate	Q > 30 rate
WT-11	135,774,224	13,713,196,624	52.44%	0.05%	92.58%
WT-21	99,548,134	10,054,361,534	52.97%	0.11%	95.44%
WT-22	94,567,544	9,551,321,944	52.18%	0.11%	95.66%
WT-23	70,231,846	7,093,416,446	51.09%	0.17%	91.85%
5xFAD-1	90,584,572	9,149,041,772	48.79%	0.04%	92.89%
5xFAD-2	92,736,068	9,366,342,868	48.50%	0.04%	92.91%
5xFAD-3	102,258,980	10,328,156,980	48.22%	0.05%	93.18%
5xFAD-21	82,518,452	8,334,363,652	50.84%	0.13%	92.06%

The sequencing reads with low quality were trimmed using the Trimmomatic (Bolger et al., 2014). To quantify the expression levels of mRNAs and lncRNAs, we used two independent analysis pipelines and intersected the results as shown in our previous article (Yoon et al., 2019). In the first approach, the trimmed sequences were aligned into the mouse genome (mm10) using the STAR aligner (Dobin et al., 2013), while the Cuffnorm was used to calculate normalized values of fragments per kilobase of transcript per million mapped reads (FPKM) based on GENCODE annotation (Release M17, GRCm38.p6; Harrow et al., 2006; Trapnell et al., 2012). Those transcripts with average FPKM values less than 1 or those not detected in any sample were removed from the further analyses. To select the transcripts with a significantly different expression between the wild type and 5xFAD groups, the *t*-test was used. In the second approach, the Salmon tool was used to quantify expression levels of transcripts (Patro et al., 2017), while the edgeR package (Robinson et al., 2010) was used to select the transcripts with significantly differing expressions between wild type and 5xFAD groups. The results from these two pipelines were intersected, and only those transcripts with significantly different expressions in both approaches were selected for further analyses.

The unsupervised hierarchical clustering was performed with Cluster 3.0 (de Hoon et al., 2004) and the results were visualized using Java Treeview (Saldanha, 2004). For the clustering, FPKM values were log-transformed, and the genes and arrays were median-centered and normalized. Complete linkage analysis was used to examine hierarchical clustering using the centered-correlation method. Those mRNAs and lncRNAs with differential expressions between the wild type and 5xFAD groups as selected from each volcano plot were used for the clustering.

### Functional Analysis of mRNAs

For gene ontology (GO) enrichment analysis, we sorted the genes based on their expression differences between wild type and 5xFAD groups. Among them, the top 10% genes with higher expressions in the pineal gland from the 5xFAD group compared to the wild type group were selected for the GO analysis of the Molecular Signatures Database (Liberzon et al., 2011; The Gene Ontology Consortium, 2017). For the same group of genes, functional annotation clustering was performed using the Database for Annotation, Visualization and Integrated Discovery (DAVID) tool (Huang da et al., 2009).

### Analysis of circRNA Expression

To detect the sequencing reads containing the back-splicing junction, the DCC algorithm was used (Cheng et al., 2016). Among the results, the exonic circRNAs, which are composed of only exonic sequences of host genes, were selected. The expression count for each circRNA was normalized by the count of total circRNA reads of each sample. To select circRNAs with differential expression between the wild type and 5xFAD groups, only circRNAs with average read numbers greater than 5 were used. To construct the regulatory network between circRNAs and miRNAs, we further selected the circRNAs with fold changes between the wild type and 5xFAD group of more than 2 and those with normalized expression counts higher than 10 among the differentially expressed circRNAs selected above.

### Prediction of miRNAs Targets

The TargetScan algorithm was used to predict the miRNA binding sites in the circRNA sequences (Agarwal et al., 2015). Among the miRNAs discovered in mice, we only used those that were “highly conserved” or “conserved” according to the annotation in the TargetScan database. After running the TargetScan to predict the miRNA binding sites in circRNAs, we only selected the targets with an “8mer site” or a “7mer-m8 site.” To draw the miRNA-circRNA interaction network, only the miRNAs with more than two binding sites in the circRNAs were used.

### Verification of the Circular Structure of circRNAs

Total RNA was treated with RNase R (Epicentre) for 20 min at 37°C to remove linear RNAs, and the enzyme was inactivated at 95°C for 3 min. The remaining RNAs were reverse-transcribed and amplified by PCR. The sequences of the PCR product were identified by Sanger sequencing to confirm the expected back-splice junctions of circRNAs. The list of PCR primers is included in Supplementary Table S4.

## Results

### Transcriptome Analysis of Pineal Gland From Mouse AD Model

**Figure 1:**
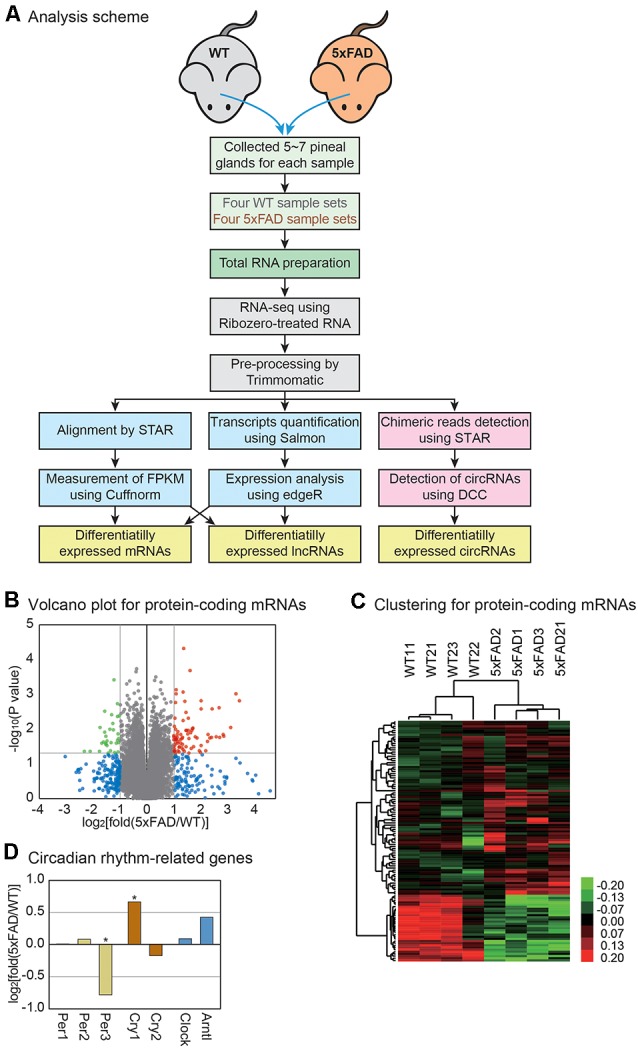
Transcriptome analysis of the mouse pineal gland with Alzheimer’s disease (AD). **(A)** Analysis scheme. The pineal glands were separated from the wild type and 5xFAD mice, and total RNAs were extracted for RNA sequencing analysis. The sequencing data were analyzed by two different pipelines, STAR-Cuffnorm and Salmon-edgeR, to identify differentially expressed mRNAs and lncRNAs. To quantify the circRNAs, chimeric reads were detected by STAR and circRNAs were measured by DCC. See the “Materials and Methods” section for details. **(B)** Volcano plot for protein-coding mRNAs. Those genes with *P*-value < 0.05 and those with a change greater than 2-fold are indicated by colored dots. **(C)** Clustering for protein-encoding mRNAs. For the selected genes from **(B)**, unsupervised hierarchical clustering was performed. The color bar represents the expression differences between the wild type and 5xFAD samples. Note that each group clustered together properly. **(D)** The expression level change of genes related to the circadian rhythm. *P*-value was calculated using a one-tailed *t*-test (* > 0.05).

The pineal gland produces melatonin, which has an important role in maintaining circadian rhythm. In addition, melatonin protects the neurons in AD by working as an antioxidant. However, no study to date has analyzed the transcriptome profile of the pineal gland from AD in part due to the difficulty in obtaining a sufficient amount of sample. To identify the protein-coding or noncoding RNAs associated with the physiology of the pineal gland of AD, we prepared the pineal gland from wild type and 5xFAD mice, a model of AD that accumulates high levels of Aβ_42_. We performed RNA sequencing for the total RNA and selected only the high-quality sequencing reads using Trimmomatic (Figure 1A; see the “Materials and Methods” section; Bolger et al., 2014).

In our previous study, we combined the results from two different pipelines for RNA sequencing analysis to increase the reliability of the expression measurement (Figure 1A; see the “Materials and Methods” section; Yoon et al., 2019). In the first pipeline, the sequences from each sample were aligned to the mouse genome by STAR, while the expression level of each gene was measured by Cuffnorm (Trapnell et al., 2012; Dobin et al., 2013). Then, the expression difference between the wild type and 5xFAD groups was calculated. In the second method, the expression of each transcript was quantified by Salmon, while the difference in the expression level was calculated using edgeR (Robinson et al., 2010; Patro et al., 2017). The results from the two approaches were compared (Supplementary Table S1) and only the genes that were changed in both analyses were used in further studies (Figure 1).

### Analysis of mRNA Changes in the Pineal Gland With AD

For the analysis of protein-encoding mRNAs, we first selected the mRNAs whose expressions differed between the wild type and 5xFAD groups. There were 81 genes with significantly increased expression and 31 genes with significantly decreased expression (Figure 1B). The unsupervised hierarchical clustering for those genes showed clear separation of the sample into the wild type and 5xFAD groups, respectively (Figure 1C).

Because the pineal gland is the central organ governing circadian rhythm, we investigated the expressions of the genes related to this process. Among the genes, we noticed a lower expression of period circadian clock 3 (Per3) and a higher expression of cryptochrome 1 (Cry1) in the pineal glands of 5xFAD mice compared to those of wild type mice (Figure 1D). It was reported that Per3 and Cry1 regulated circadian feedback loops to generate 24-h oscillations (Reppert and Weaver, 2002). These clock genes are expressed in the pineal gland and provide information to other central and peripheral structures (Fukuhara et al., 2000; Takekida et al., 2000; Yamazaki et al., 2000). Therefore, there might be an alteration in the circadian regulation in the pineal gland of AD.

**Figure 2:**
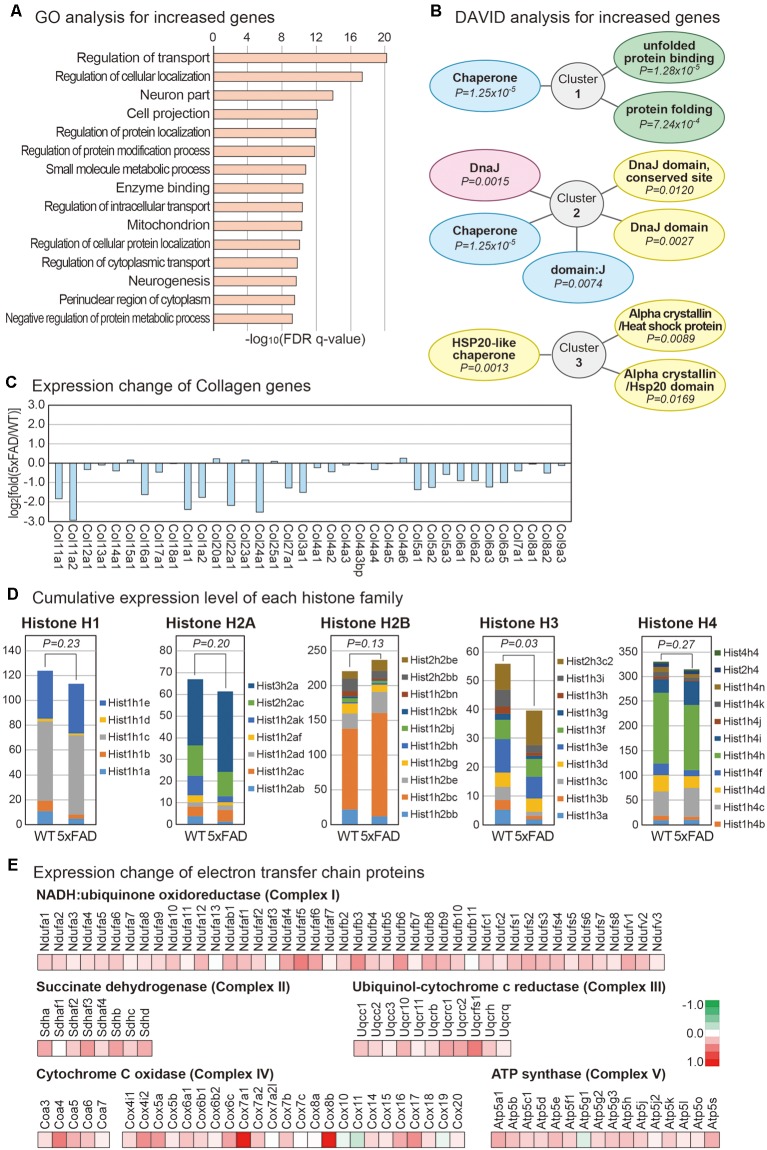
Analysis of mRNAs changes in the mouse pineal gland with AD. **(A)** Gene ontology (GO) analysis of increased protein-coding mRNAs in the pineal gland from 5xFAD vs. wild type mice. The top 15 GO terms based on the false discovery rate (FDR) *q*-value are shown. **(B)** Database for Annotation, Visualization and Integrated Discovery analysis for increased protein-coding mRNAs. The top three enriched clusters are visualized. **(C)** Expression changes in the collagen genes are shown. **(D)** Expression of histone gene groups. The sum of fragments per kilobase of transcript per million mapped reads value for the members of each histone group was calculated. A one-tailed *t*-test was applied to calculate the *P*-value. **(E)** Expression changes for the members of the electron transfer chain in mitochondria. The expression changes for members from each mitochondria complex between the pineal glands from the wild type and 5xFAD groups are shown.

To identify the cellular pathways affected in the pineal gland of AD, we first performed the GO analysis for increased genes in the 5xFAD vs. wild type group (The Gene Ontology Consortium, 2017). The most significantly enriched terms included those related to cellular transport and protein localization (Figure 2A). Many studies suggested that the process related to protein sorting is defective in the neurons of AD (Small and Gandy, 2006). Interestingly, the expression of myosin genes was globally decreased in the 5xFAD group compared to the wild type group (Supplementary Figure S1A). Thus, the processes related to the movement of cellular molecules including proteins might also be dysregulated in the pineal gland of AD. We also performed a functional analysis of increased genes using the DAVID functional annotation tool (Huang da et al., 2009). Interestingly, all three of the most highly enriched clusters were related to the cellular pathway of protein folding (Figure 2B). In diverse human diseases of the brain including AD and Parkinson’s disease, misfolded proteins are commonly observed (Chiti and Dobson, 2017). In the brain of AD, Aβ is highly accumulated, leading to a problem in the protein folding process within the neurons. Therefore, a similar defect in protein folding occurs in the pineal gland of AD. We also noticed the global decrease in collagen genes (Figure 2C). Because collagen has a protective role for neurons, its decreased expression might affect the impaired function of the pineal gland in patients with AD (Cheng et al., 2009).

Analysis of histone gene expression revealed that the expression of overall histone genes is decreased in the pineal gland of 5xFAD mice compared to wild type mice (Supplementary Figure S1B). Interestingly, the FPKM values of most decreased genes were low, while those of some increased genes were relatively higher. This expression change resulted in a non-meaningful difference overall when we considered the expression level of each histone group by combining the FPKM for each histone class. Thus, there was no difference in the total amount of most histone classes, although there was a redistribution on histone expression in each gene class. However, the overall transcripts dosage of histone H3 class decreased significantly in the 5xFAD group compared to the wild type group (Figure 2D). Histone influences overall DNA metabolism, such as DNA repair (Singh et al., 2009), and histone dosage controls DNA damage sensitivity in a checkpoint-independent manner in cells (Liang et al., 2012). Therefore, the dysregulation of histone dosage may have a role in the cellular physiology of the pineal gland of AD.

Strikingly, the expression of nearly all components of the electron transfer chain increased in the 5xFAD group compared to the wild type group (Figure 2E). The electron transfer chain creates a proton gradient across the inner membranes of mitochondria, driving the synthesis of adenosine triphosphate (ATP). One study showed that ATP is involved in the inhibition of melatonin synthesis in the rat pineal gland (Souza-Teodoro et al., 2016). Thus, it would be interesting to study whether the increased expression of electron transfer chain complexes might affect the physiology of AD pineal glands.

### Analysis of lncRNAs Change in the Mouse Pineal Gland With AD

**Figure 3:**
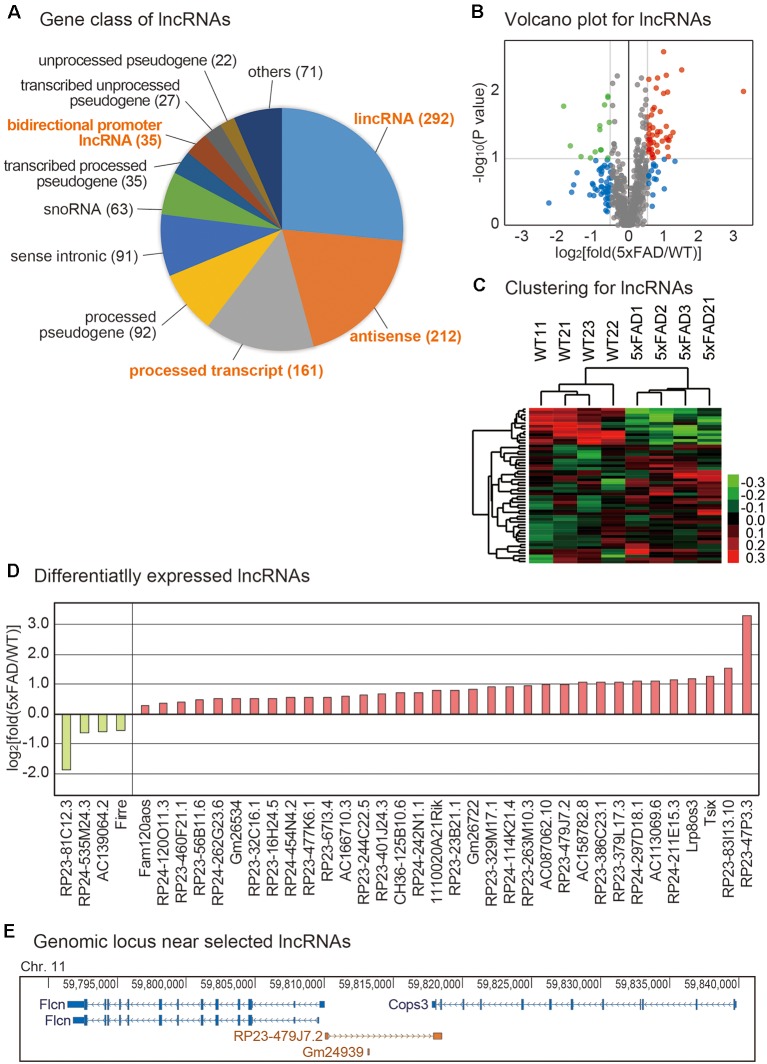
Analysis of lncRNAs changes in the mouse pineal gland with AD. **(A)** Distribution of identified lncRNAs from pineal gland samples based on the GENCODE annotation. The number of identified lncRNAs for each lncRNA class is shown in parentheses. **(B)** Volcano plot for lncRNAs. Those genes with *P*-value < 0.1 and those with expression changes >50% in the 5xFAD group compared to the wild type group are indicated with colored dots. **(C)** Clustering for lncRNAs. For the selected lncRNAs from **(B)**, unsupervised hierarchical clustering was performed. The color bar representing the expression difference between the wild type and 5xFAD groups is shown. **(D)** List of differentially expressed lncRNAs between the pineal gland from the wild type and 5xFAD groups. **(E)** Genomic information near the locus of lncRNA RP23-479J7.2 and its near protein-coding genes, Flcn and Cops3. The genomic information data were downloaded from the Genome Browser (Kent et al., 2002).

Although lncRNAs are involved in diverse aspects of cellular processes, no study to date has profiled the lncRNAs in the pineal glands of AD. To identify lncRNAs that are dysregulated in the pineal gland of AD, we analyzed the expression level of lncRNAs from the RNA sequencing data produced above. Diverse types of lncRNA genes exist in the genome as annotated in GENCODE (Harrow et al., 2006). Among those classes, we selected long intergenic noncoding RNA (lincRNA), antisense RNA, the processed transcript, and the bidirectional promoter lncRNA for further analysis (Figure 3A) because these classes are more likely to be transcribed as RNA transcripts than other classes such as pseudogenes. Among them, we selected the lncRNAs with a significant difference in their expression between the pineal glands of the wild type and 5xFAD groups (Figure 3B, Supplementary Table S2). The unsupervised clustering analysis for those selected lncRNAs showed a clear separation of samples into the wild type and 5xFAD groups (Figure 3C).

Among the differentially expressed lncRNAs, we selected the lncRNAs with highly significant expression changes (Figure 3D). Because the genes closely located in the genomic location have a higher tendency to be influenced by a common signaling pathway (Lee and Sonnhammer, 2003; Michalak, 2008), we investigated the protein-coding gene with a known function related to neuronal processes near each lncRNA. For example, the most highly increased lncRNA, RP23-47P3.3, and the most highly decreased lncRNA, RP23-81C12.3, have no neighboring protein-encoding genes in their vicinity. However, RP23-479J7.2, which we confirmed its expression change (Supplementary Figure S2A) had its neighboring gene, Cops3, which resided in the genomic locus commonly deleted in patients with Smith-Magenis syndrome (Figure 3E; Potocki et al., 2000). Thus, it is possible that they are regulated under the common signaling pathway.

### Analysis of circRNAs Change in the Mouse Pineal Gland With AD

**Figure 4:**
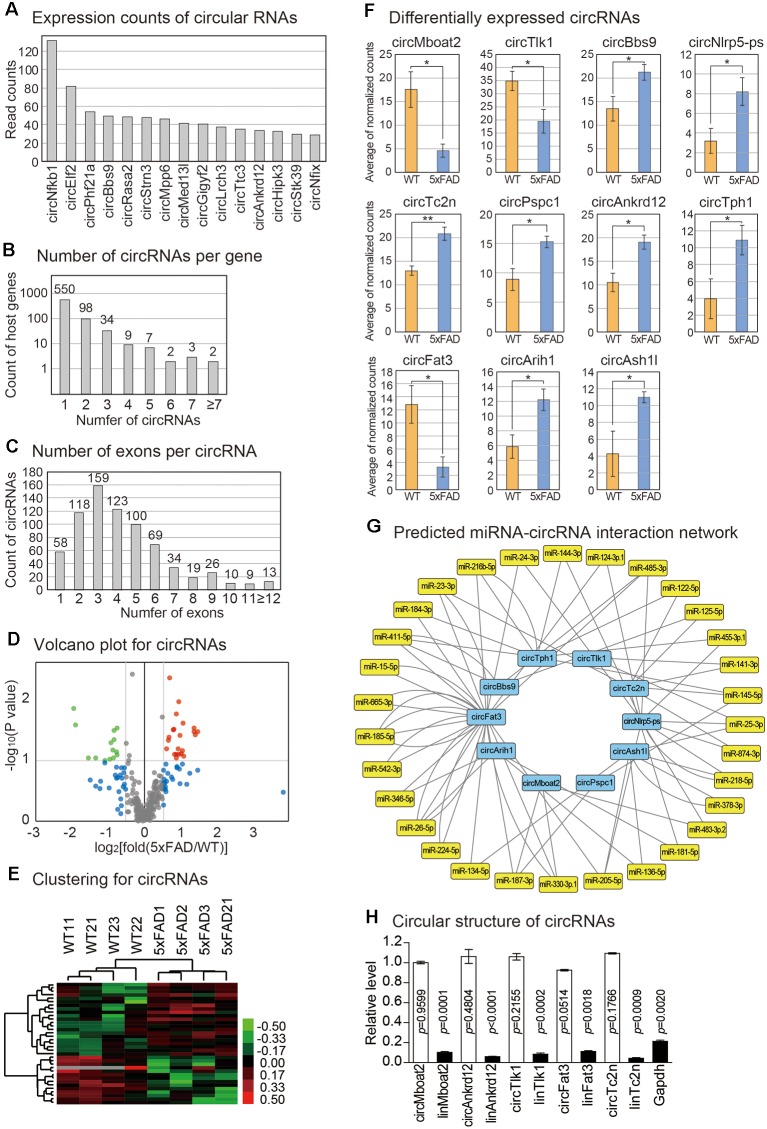
Analysis of circRNA changes in the mouse pineal gland with AD. **(A)** Expression counts of 15 highly expressed circRNAs in the pineal glands. The average read counts among the wild type and 5xFAD samples were calculated. **(B)** Distribution of the number of circRNAs produced from each host gene. **(C)** Distribution of the number of exons used to produce each circRNA. **(D)** Volcano plot for circRNAs. Those genes with values of *P* < 0.1 and those with expression changes >50% in the 5xFAD group compared to the wild type group are indicated. **(E)** Clustering of circRNAs. For the selected circRNAs from **(D)**, unsupervised hierarchical clustering was performed. The color bar representing the expression difference between wild type and 5xFAD is shown. **(F)** Differentially expressed circRNAs between the pineal gland from the wild type and 5xFAD groups. A one-tailed *t*-test was applied to calculate the *P*-value (* < 0.05, ** < 0.01). **(G)** The circRNAs-miRNAs regulatory network. The regulatory relationship was predicted by the existence of a miRNA binding site in the circRNA sequence. **(H)** Confirmation of the circular structure of circRNAs by RNase R treatment. The data in Supplementary Figure S2B were used for the quantitation. The white bars indicate circRNAs while the filled bars are linear RNAs. Expression change of each circRNA between untreated and RNase R-treated samples was calculated. *P*-value was calculated with a two-tailed *t*-test.

CircRNAs are emerging as important regulators of cellular processes, mainly through the suppression of miRNA function. No study examined the circRNAs in any aspect of the pineal gland. By analyzing the back-splicing junction from the RNA sequencing reads based on the DCC algorithm, we calculated the expression level of circRNAs in the pineal glands of the wild type vs. 5xFAD mice (Figure 1A; see the “Materials and Methods” section; Cheng et al., 2016). We identified a total of 744 circRNAs expressed in our samples (Supplementary Table S3), and some circRNAs showed high expression levels (Figure 4A). The circRNA produced from the locus of the Nfkb1 gene showed the highest expression level. When we analyzed the number of circRNAs produced from each gene locus, we found that most host genes produced only a single type of circRNA, although some produced diverse circRNAs with a different combination of host gene exons (Figure 4B). Moreover, one to six exons were combined to compose circRNAs with three exons as the highest frequency (Figure 4C). In our previous analysis of the circRNAs in the brain cortex, the combination of three exons was also the most frequent choice for the production of circRNA (Yoon et al., 2019). Therefore, there may be a similar principle to produce circRNA by combining exons between the brain cortex and the pineal gland.

We selected the circRNAs with significant expression differences between the pineal glands of wild type and 5xFAD mice (Figure 4D). We also confirmed that the samples that we analyzed were clustered properly from unsupervised hierarchical clustering (Figure 4E). Among the differentially expressed circRNAs, we further selected 11 circRNAs whose expression differences between the wild type and 5xFAD group were more significant or whose expression levels were high in the pineal gland (see the “Materials and Methods” section; Figure 4F). To predict the miRNAs that could be under the control of these circRNAs, we utilized the TargetScan algorithm to predict the miRNA binding sites in the circRNA sequences (Agarwal et al., 2015). Indeed, there were many miRNA binding sites predicted in the circ-RNAs sequence (Figure 4G). Therefore, the altered expression of these circRNAs in the pineal gland of AD may exert their roles by suppressing the predicted miRNAs. We confirmed the circular structure and expression changes of five randomly selected circRNAs (Figure 4H, Supplementary Figure S2). All of the selected circRNAs had a circular structure in the cells as confirmed by their resistance to RNase R, an enzyme that degrades linear RNAs, and their back-splicing junction prediction was verified by Sanger sequencing.

## Discussion

In mammals, the sympathetic innervation of the pineal gland contributes to the transduction of environmental light information into a hormonal signal such as melatonin. A previous study analyzed the light-induced transcriptome of the pineal gland of zebrafish and showed that miR-183 affects the mRNA level of aanat2, the key enzyme in melatonin synthesis (Ben-Moshe et al., 2014). It was also reported that miR-483 regulated the synthesis of melatonin by inhibiting Aanat in the pineal glands of rats (Clokie et al., 2012). Another study identified lncRNAs that are differentially expressed between day and night in the pineal glands of rats (Coon et al., 2012). However, no study has analyzed the transcriptome in the pineal gland of AD. Therefore, here we scrutinized the differential expression of diverse RNA types including protein-encoding RNAs, lncRNAs, and circRNAs in the pineal gland of AD.

The circadian rhythm is regulated by the oscillations of the transcriptional-translational negative feedback system. During sleep, brain and muscle ARNT-like-1 (BMAL1) heterodimerizes with circadian locomotor output cycles gone kaput (CLOCK), and the CLOCK/BMAL1 heterodimer regulates the transcription of period (Per) and cryptochrome (Cry; Ko and Takahashi, 2006). Per loss has been known to regulate the transcriptional activity of CLOCK (Hardin and Panda, 2013) and accelerates cell death and motor dysfunction (Krishnan et al., 2012). The loss of Per3 protein in particular among the period genes inhibits the Per1/2 stabilization and induces circadian rhythm disruptions (Zhang et al., 2016). In addition, cryptochrome (Cry1/2) suppresses CLOCK and BMAL1 expressions (Reppert and Weaver, 2002). The rhythm of clock gene expression in the pineal gland is aberrantly regulated in AD patients; subsequently, the aberrant circadian regulation leads to memory deficits in AD in relation to the AD development (Coogan et al., 2013). In our data, the lower Per3 expression and higher Cry1 expression might lead to the aberrant circadian rhythm based on this previous evidence.

In our analysis, the expression of most components of the electron transfer chain increased in the pineal gland of AD. These proteins comprise the main complexes in the mitochondria to produce the cellular energy source, ATP, a known co-transmitter of noradrenaline in the pineal gland (Mortani Barbosa et al., 2000) and boost β1-adrenergic-induced N-acetylserotonin synthesis by triggering P2Y1 receptors (Ferreira and Markus, 2001). Recent studies demonstrated that ATP suppressed melatonin synthesis by the pineal gland (Souza-Teodoro et al., 2016). Furthermore, ATP is one of the damage-associated molecular pattern molecules (Di Virgilio, 2007), and a high amount of it is released under stress conditions (Carta et al., 2015). Thus, the increased expression of the members of the electron transfer system in 5xFAD mice may increase ATP production and can result in pineal gland impairment.

Collagens comprise a large family of proteins involved in a variety of functions ranging from the formation of fibrillary networks of the extracellular matrix to synapse formation. One study revealed that the increase in collagen VI expression in the brains of hAPP mice is a neuroprotective response (Cheng et al., 2009). Other collagen genes provide guidance for axon outgrowth (Taylor et al., 2018), help motor neurons to innervate (Kowa et al., 2004), and regulate Aβ peptide formation during the progression of AD (Osada et al., 2005). Because of these important roles of collagen in neurons, we assume that the loss of collagen in the AD pineal gland is linked to pineal gland dysfunction.

No study to date has analyzed the transcriptome of the pineal gland in an AD model. In this study, we identified diverse lncRNAs and circRNAs that were differentially expressed in the pineal gland in the 5xFAD group vs. the wild type group (Figures 3, 4). Because many lncRNAs work by regulating their near protein-encoding genes, we investigated the genomic locus of lncRNAs and searched the genes near them. Among the lncRNAs, RP23-479J7.2 had a near protein-coding gene Cops3, whose genomic locus is commonly deleted in patients with Smith-Magenis syndrome (Potocki et al., 2000). Smith-Magenis syndrome is a syndrome that features mental retardation and sleep disturbances as well as problematic daytime behavior (De Leersnyder, 2006). This problem was linked to abnormal circadian secretion of melatonin. Thus, a future study must elucidate the detailed role of RP23-479J7.2 in this disease.

We also identified many altered circRNAs in the pineal gland of an AD model. Because the circRNAs mainly work by binding miRNAs and suppressing their function, we predicted the miRNAs with binding sites in circRNA sequences (Figure 4G). Among the identified miRNA-circRNA pairs, circMboat2 and circNlrp5-ps were predicted to be regulated by miR-483, which was shown to suppress Aanat, the enzyme critical for melatonin synthesis (Clokie et al., 2012). Therefore, these circRNAs may be involved in the regulatory network of pineal gland function such as melatonin synthesis. Further studies including the profiling of miRNAs are essential to identify the role and working mechanism of noncoding RNAs in the pineal gland of AD.

This is the first study to profile the transcriptome of the pineal gland from the AD model. We expect that our study will be helpful for the researchers interested in the gene expression change of the pineal gland with AD. Although we identified diverse RNAs differentially expressed between the pineal glands of wild type and 5xFAD mice, it needs to be noted that the experiment was performed at a 1-time point (ZT0.5). Therefore, there is a possibility that the gene expression changes could be the result of a shift in the circadian rhythm of the AD model. In a future study, large-scale profiling of the transcriptomes with various time points of the day will be required for a more comprehensive understanding of the change in the AD pineal gland.

## Data Availability Statement

The raw sequencing data and processed data with FPKM values are available through the Gene Expression Omnibus database under accession number GSE129586 (Barrett et al., 2013).

## Ethics Statement

The animal study was reviewed and approved by Animal Ethics Committee at Chonnam National University.

## Author Contributions

KN and JS prepared the samples for the analysis. Y-KK performed the bioinformatics analyses. GY confirmed the expression of the selected genes by biochemical analysis. JS and Y-KK wrote the manuscript.

## Conflict of Interest

The authors declare that the research was conducted in the absence of any commercial or financial relationships that could be construed as a potential conflict of interest.

## Supplementary Material

FIGURE S1 Expression of protein-coding genes of the selected gene groups. **(A)** Expression changes of the myosin genes. **(B)** Expression changes of the histone genes. A one-tailed *t*-test was applied to calculate the *P*-value (* < 0.05, ** < 0.01, *** < 0.001).

FIGURE S2 Measurement of lncRNA and circRNAs level and confirmation of the circular RNA structure. **(A)** Measurement of lncRNA and circRNAs by PCR. The expression change of the lncRNA RP23-479J7.2 in Figure 3E and five randomly selected circRNAs in Figure 4F were measured. *P*-values were calculated by a two-tailed *t*-test. **(B)** Confirmation of the circular structure of circRNAs by RNase R treatment. This data was used for the quantitation in Figure 4H.

TABLE S1 Expression of protein-coding mRNAs.

TABLE S2 Analysis of lncRNA expression.

TABLE S3 The read counts of circRNAs measured by STAR-DCC algorithm.

TABLE S4 Primers used in this study.
