# Supplementary figures and images for: Transcriptome Analysis of Pineal Glands in the Mouse Model of Alzheimer’s Disease
Source: Front Mol Neurosci. 2020 Jan 9;12:318. doi: 10.3389/fnmol.2019.00318 (PMC6962250; doi:10.3389/fnmol.2019.00318)

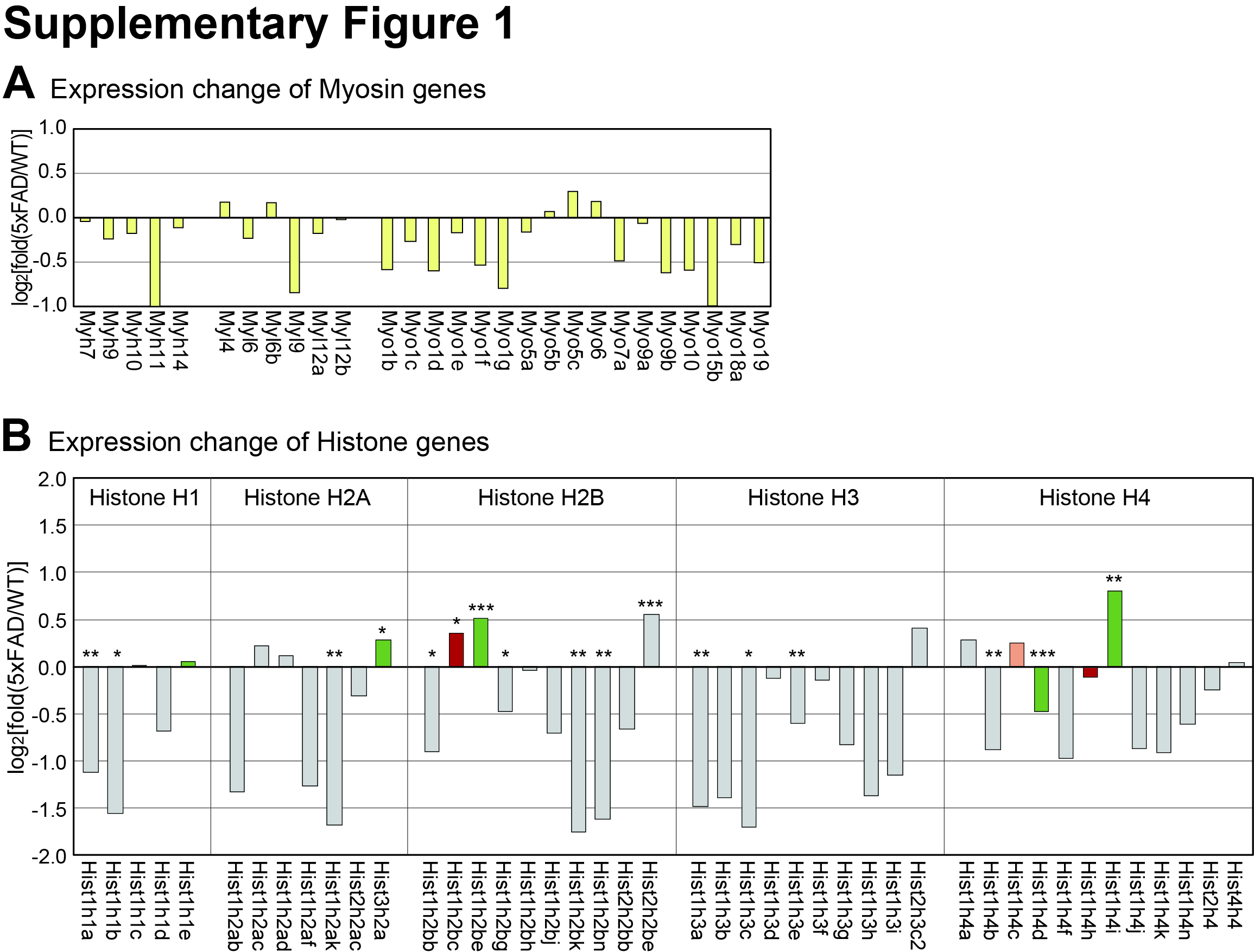

Supplement: FIGURE S1 — Expression of protein-coding genes of the selected gene groups. (A) Expression changes of the myosin genes. (B) Expression changes of the histone genes. A one-tailed t-test was applied to calculate the P-value (* < 0.05, ** < 0.01, *** < 0.001). [file Image_1.JPEG]

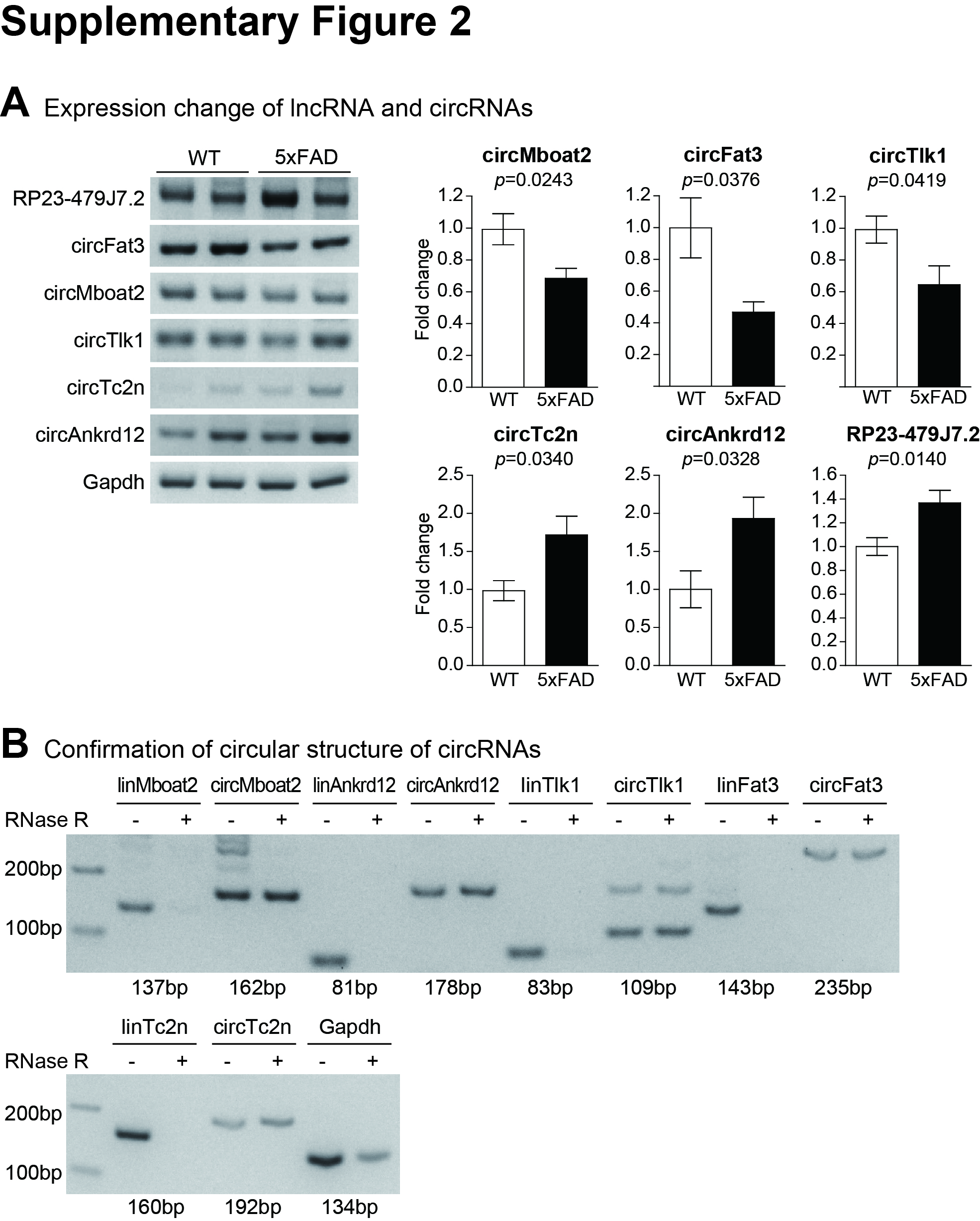

Supplement: FIGURE S2 — Measurement of lncRNA and circRNAs level and confirmation of the circular RNA structure. (A) Measurement of lncRNA and circRNAs by PCR. The expression change of the lncRNA RP23-479J7.2 in Figure 3E and five randomly selected circRNAs in Figure 4F were measured. P-values were calculated by a two-tailed t-test. (B) Confirmation of the circular structure of circRNAs by RNase R treatment. This data was used for the quantitation in Figure 4H. [file Image_2.JPEG]
